# Assessment of influencing factors of hospitalization expenses for Crohn's disease patients: Based on LASSO and linear mixed model

**DOI:** 10.3389/fpubh.2022.925616

**Published:** 2022-09-09

**Authors:** Li Wu, Zhijie Lv, Linjing Lai, Penglei Zhou

**Affiliations:** ^1^Center of Clinical Evaluation, The First Affiliated Hospital of Zhejiang Chinese Medical University, Hangzhou, China; ^2^Center of Clinical Evaluation, Zhejiang Provincial Hospital of Traditional Chinese Medicine, Hangzhou, China; ^3^Department of Pharmacy, Sir Run Run Shaw Hospital, Zhejiang University School of Medicine, Hangzhou, China

**Keywords:** Crohn's disease, LASSO, linear mixed model, hospital expenses, influencing factors

## Abstract

**Aims:**

Crohn's disease (CD) is a global disease that is dramatically increasing. This study aimed to identify the primary drivers of hospitalization expenses for CD patients to provide guidance on the allocation and control of health care costs.

**Methods:**

This study retrospectively collected the homepage data of the electronic medical records of CD patients in two tertiary hospitals in Zhejiang Province, China, from January 2016 to December 2021. The influencing factors of hospitalization expenses for CD were analyzed. A linear mixed model with least absolute shrinkage (LASSO-LMM) was used to develop a predictive model for hospitalization expenses for CD patients.

**Results:**

A total of 4,437 CD patients were analyzed in this study. CD patients' age, length of hospital stay, admission route, comorbidities, and main treatment were found to be statistically significant variables for CD patients' hospitalization expenses. The AIC and BIC of LASSO-LMM model were 319.033 and 306.241, respectively. Patients who were older, had a longer hospital stay, and had comorbidities had higher hospitalization expenses. The hospitalization expenses of outpatients were lower than those of emergency patients. The weight of surgical treatment was the highest among three treatments (0.602).

**Conclusions:**

Identifying and examining factors that influence hospitalization expenses for CD patients can help to control healthcare expenditures. Treatment mode was the most important impact on CD hospitalization expenses. Medical security departments can consider implement personalized and precise hospitalization expense compensation scheme base on LASSO-LMM prediction model in the future.

## Introduction

Crohn's disease (CD) is a chronic inflammatory disease with a relapse-remission process, showing a different disease spectrum (1). It is one of the most prevalent inflammatory bowel diseases (IBD).The etiology of CD is still unknown, but genetic, immune, and environmental factors increase the risk of onset and progression of the disease (2).CD is characterized by skipping intestinal injury anywhere in the gastrointestinal tract and involves chronic, recurrent wall inflammation, which can cause chronic abdominal pain, diarrhea, obstruction and / or perianal injury (2, 3).

In the past, CD was commonly seen in Western regions such as North America and Northern Europe, with the highest prevalence rates being in Europe (322 per 100 000), Canada (319 per 100 000) and the USA (214 per 100 000) (2). The incidence of CD has risen globally since the beginning of the 21st century, as rapidly reported in emerging industrialized countries in Asia, Africa, and South America (4). In China, a retrospective analysis showed that the incidence of CD is on the rise. It is estimated that the prevalence of CD in China has reached 1.4 per 100,000 person-years (5), which is the highest in Asia (6).

The increasing number of CD patients has caused a corresponding increase in CD treatment costs. It is well established that CD's economic burden is unbalanced due to the heterogeneity of disease processes and complications. A systematic review estimated that the pooled mean hospitalization cost per patient per year for CD patients was €2004.83. The main contributors toward total CD health expenditure were biologics (€5554.58) and medications (€3096.53), followed by hospitalization (€2004.83) and surgery (€1883.67) (7). In China, the economic burden of CD was 54,246 *Yuan*, which exceeds the cost of other chronic diseases such as diabetes (8). A web-based study on the financial burden and medical service availability of IBD patients in China showed that Chinese IBD patients had huge economic burdens and difficulties in acquiring health care, which enlarged their economic anxiety and inevitably affected the outcomes of their disease (9). Given the intermittent periods of hospitalizations, surgery, and pharmacotherapy, CD patients go through the management of flare-ups and disease complications during their lifetime, CD accounts for a substantial amount of costs to health care systems and society in general (10, 11).

The traditional models for screening the influencing factors of hospitalization expenses include multiple linear regression model (12, 13), partial least square structural equation model (14), data mining method (15, 16) and others. As hospitalization expenses are affected by patients' own characteristics and treatment methods, they often present a multi-level distribution. The linear mixed model (LMM), also known as a multi-level model, contains both fixed effects and random effects. It can not only deal with the relationship between different levels of factors, but also explore the impact of various levels of influencing factors on hospitalization expenses. However, there are many factors affecting the hospitalization expenses of CD patients, and certainly not all of these factors play a key role in hospitalization expenses, and some factors may even interfere with the changes of hospitalization expenses. Recently, with the development of big data, a method of factor selection through coefficient compression has attracted increasing attention from scholars. A past study (15) used a Random Forest (RF) model and Least Absolute Shrinkage and Selection Operator (LASSO) Regression to predict the medical expenses of chronic renal failure patients. Another study (17) explored four machine learning models, including ordinary least squares linear regression, regularized regression, gradient booster, and recurrent neural network, to predict health care expenditures. However, the research on the prediction and influencing factors of the hospitalization expenses in CD patients has, to the current authors' knowledge, not been reported.

The hospitalization expense has become a direct financial burden for patients, it also concerned by medical insurance departments. The China Medical Insurance Administration is currently implementing a new medical reform policy, and has introduced medical insurance payment methods for disease diagnosis-related groups (DRG) and diagnostic intervention packages (DIP) to address the burden of medical expenses (15). These payment methods have comprehensively considered China's medical insurance policies, innovatively integrated the characteristics of various diseases, which puts forward higher requirements for medical insurance payment standards. Therefore, the linear mixed model with least absolute shrinkage (LASSO-LMM) presents a new prospective for rational allocation and control of hospitalization expenditure, particularly in the era of big data.

This study selected CD inpatients from two tertiary hospitals in Zhejiang Province as the research participant. Our study intends to use the method of LASSO-LMM to predict individuals' hospitalization expenses and assess associated factors according to data from the homepage of CD patients' hospital records. This is so we can realistically evaluate the influencing factors of hospitalization expenses and provide a reference point for future health policies and medical expense reimbursement systems related to CD patients in China.

## Materials and methods

### Data source

This study retrospectively collected the homepage data of the electronic medical records of CD patients in two tertiary hospitals in Zhejiang Province from January 2016 to December 2021. The data was used to identify patients with CD through the International Diagnosis Classification Code, 10th Revision (ICD-10). The first three code K50 in ICD-10 represents the classification of CD. In this study, a total of 20,795 hospitalized medical records were retrieved with K50 as the main diagnostic code. Guided by the relevant reports (15, 18, 19), the data set used in our research included patient details such as age, sex, marital status, medical payment method, admission route, length of hospital stay, comorbidities, major procedures, main treatment, rehospitalization plan within 31 days of discharge, and discharge method. The target variable was total hospitalization expenses. All expenditure variables were converted to 2016 Chinese *Yuan* (¥) using the Consumer Price Index.

To protect patients' privacy, their identities were concealed, and only medical record numbers were used. This study was conformed with the “Ethics review methods for biomedical research involving humans” promulgated by the Ministry of Health of The People's Republic of China and was performed according to the principles of the Helsinki Declaration.

### Data preprocessing

In order to ensure the reliability of the results, according to the purpose of this study, we excluded cases where patients left the hospital on the first day, there was missing data, variables with small sample sizes (*n* < 10), and hospitalization expenses outliers.

To eliminate the influence of the unit difference between different variables affecting hospitalization expenses, the normal transformation function $x^{*}=\frac{x-\mu }{\sigma }$was used to standardize continuous variables. Where μ and σ were the mean and standard deviation of the variable, respectively. The data value > μ+3σ or < μ−3σ were considered outliers and removed.

Since age, length of stay, number of comorbidities, and hospitalization expenses were continuous variables, by calculating skewness and kurtosis, none of these variables followed a distribution distribution. Thus, these continuous variables were grouped, statistically described as counts and proportions, and the median and quartile of the total medical expenses were calculated for each group.

### Statistical analysis

Continuous variables with normal distribution were expressed as mean ± standard deviation (SD). Continuous variables with non-normal distribution or ordinal variables were expressed as median (the lower four quantile, the upper four quantile). Categorized variables were summarized as counts and proportions. Mann-Whitney *U* test or Kruskal-Wallis *H* test was used to compare hospital expenses for each group. Then, linear mixed-effects model with LASSO (LASSO-LMM) was used to further explore the influencing factors of CD hospitalization expenses.

#### Least absolute shrinkage and selection operator

Least Absolute Shrinkage and Selection Operator (LASSO) is a factor screening method, which compresses variable coefficients by constructing penalty functions to solve the problem of over-fitting regression models. The basic idea of the algorithm is to minimize the sum of absolute values of regression coefficients under the constraint of a constant, and to minimize the sum of squares of residuals, so that some regression coefficients are strictly contracted to zero.


$$\begin{array}{l} {\hat{\beta }}_{lasso}=arg\min_{\beta }|Y-\overset{p}{\underset{j=1}{\sum }}X_{j}\beta_{j}{|}^{2}+\lambda \overset{p}{\underset{j=1}{\sum }}|\beta_{j}| \end{array}$$

In the formula, the penalty term $\lambda \overset{p}{\underset{j=1}{\sum }}|\beta_{j}|$ is the absolute value of the factor coefficient of hospitalization expenses, and the penalty parameter is used to control the shrinkage of the regression coefficient of the linear mixed model. When λ is large enough, it will compress the regression coefficient and make it tend to 0, so as to achieve the purpose of screening the key influencing factors of hospitalization expenses.

#### Linear mixed-effects model

Linear mixed–effects model, as an important statistical model widely used in economic, medical, financial and other fields, includes fixed effects and random effects. Suppose there are n subjects with Gaussian response{*y*_*i*_:*i* = 1, 2, …, *n*}, some important covariates *X*_*i*_ = (*x*_1_, *x*_2_, …, *x*_*q*_) are considered for the fixed effects. Denote *Z*_*i*_ = (*z*_1_, *z*_2_, …, *z*_*q*_) as a set of random effect covariates that represent individual departure from the fixed effect. The basic mixed effects model is:


$$\begin{array}{l} y_{i}=X_{i}\beta +Z_{i}b_{i}+e_{i} \end{array}$$

Where *X*_*i*_ and *Z*_*i*_ are *n*_*i*_× *p* and *n*_*i*_× *q* matrices, respectively; and *y*_*i*_, β, *b*_*i*_ and *e*_*i*_are *n*_*i*_× 1, *p*× 1, *q*× 1and *n*_*i*_× 1vectors. In the above model, β represents the fixed effects, *b*_*i*_ represents the random effects and *e*_*i*_ represents observation errors.

The above linear mixed effects model has been deeply studied in some literature (20, 21).

#### Prediction performance evaluation

The Akaike Information Criterion (AIC) and Bayesian Information Criterion (BIC) were used to evaluate the fitting effect of LASSO-LMM model. The smaller their values, the better the model.

The above statistical analyses were performed with the IBM SPSS statistics software (IBM SPSS Statistics for Windows, Version 25.0. Armonk, New York) and R version 4.1.1. All statistics were two-sided and considered significant if the *P*-value was <0.05.

## Results

### Demographic characteristics

**Figure 1:**
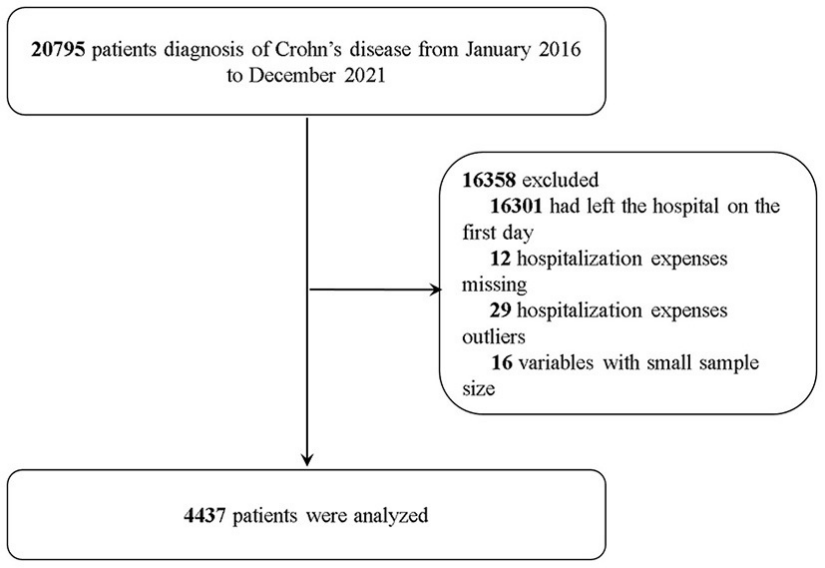
Patient selection in a study of Crohn's disease.

We ultimately identified 4,437 hospitalized patients with CD according to inclusion and exclusion criteria (Figure 1). Of the hospitalized patients, 3,001 were male (67.6%); the median (IQR) age was 34 years old (24~42), and 40.5% of patients were 19 to 30 years old. Nearly half of the patients (45.1%) were married. The majority of hospital admissions were from outpatients (95.0%), with an average length of stay of 10.89 days. There were 3,544 patients with comorbidities or complications (79.9%). A large number of patients underwent surgical or procedural treatment (71.4%). Most patients had no readmission plan within the 31 days after they were discharged (84.4%). The average hospitalization expense was 14,654 Yuan.

### Analysis of differences in hospitalization expenses of Crohn's disease

Table 1 displays hospitalization expenses of patients with CD under different clinical characteristics. The Mann-Whitney *U*-test and Kruskal-Wallis *H*-test were performed.

**Table 1:** Baseline characteristics and difference in hospitalization expenses of Crohn's disease (*N* = 4,437).

**Variables**	***N* (%)**	**Median (Q_L_, Q_U_)**	**Test statistic (*U*/*H*)**	***P-*Value**
Sex			1.851	0.064
Male	3,001 (67.6)	7,433 (4,316, 14,106)		
Female	1,436 (32.4)	7,649 (4,632, 16,727)		
Marital status			128.279	<0.001
Unmarried	1,124 (25.3)	6,147 (3,237, 11,035)		
Married	2,002 (45.1)	7,350 (4,367, 17,149)		
Other	1,311 (29.5)	8,876 (5,586, 16,380)		
Medical payment method			1.218	0.223
Medical insurance	3,533 (79.6)	7,513 (4,464, 14,982)		
Other social insurance	904 (20.4)	7332 (4,225, 14,149)		
Age, years			59.415	<0.001
<18	198 (4.5)	6,843 (3,926, 11,518)		
18~39	2,922 (65.9)	7,202 (4,123, 13,637)		
40~59	1,116 (25.2)	8,010 (4,957, 20,733)		
≥60	201 (4.5)	9,460 (5,630, 34,720)		
Admission route			4.528	<0.001
Emergency	224 (5.0)	8,970 (5,848, 24,930)		
Outpatient	4,213 (95.0)	7,386 (4,367, 14,480)		
Length of stay, day			2008.363	<0.001
2~10	3,277 (73.9)	5,798 (3,504, 8,729)		
10~20	877 (19.8)	26,808 (12,781, 44,744)		
≥21	283 (6.4)	53,483 (43,649, 64,181)		
Comorbidities			6.964	<0.001
No	893 (20.1)	6,160 (2,433, 14,686)		
Yes	3,544 (79.9)	7,750 (4,735, 14,788)		
Main treatment			2460.321	<0.001
Medical treatment	1,271 (28.6)	3,419 (1,921, 5,676)		
Procedural treatment	2,081 (46.9)	7,493 (5,245, 11,348)		
Surgical treatment	1,085 (24.5)	40,924 (16,510, 51,627)		
Readmission plan within 31 days of discharge			5.473	<0.001
No	3,747 (84.4)	7,655 (4,452, 16,786)		
Yes	690 (15.6)	6,674 (4,271, 10,387)		
Discharge method			1.417	0.156
Normal discharge	4,330 (97.6)	7492 (4,414, 14,646)		
Transfer to another hospital	107 (2.4)	8,309 (4,755, 34,099)		

The pairwise comparison results for further *post-hoc* tests were presented in Table 2.

**Table 2:** Significance results of pairwise comparison.

**Variables (pairwise comparisons between groups)**	**Test statistic**	***P*-value**	**Adj *P*-value**
**Marital status**			
Unmarried vs. married	328.141	<0.001	<0.001
Unmarried vs. other	589.605	<0.001	<0.001
Married vs. other	261.464	<0.001	<0.001
**Age, years**			
<18 vs. 18~39	−135.248	0.151	0.903
<18 vs. 40~59	−381.694	<0.001	0.001
<18 vs. ≥60	−654.070	<0.001	<0.001
18~39 vs. 40~59	−246.446	0.001	<0.001
18~39 vs. ≥60	−518.822	<0.001	<0.001
40~59 vs. ≥60	−272.376	0.006	0.033
**Length of stay**			
2–10 vs. 11–20	−1785.642	<0.001	<0.001
2–10 vs. ≥21	−2404.969	<0.001	<0.001
11–20 vs. ≥21	−619.327	<0.001	<0.001
**Main treatment**			
Medical treatment vs. Procedural treatment	−1152.644	<0.001	<0.001
Medical treatment vs. Surgical treatment	−2625.156	<0.001	<0.001
Procedural treatment vs. Surgical treatment	−1472.512	<0.001	<0.001

The Bonferroni-adjusted alpha level was used to determine whether there were significant differences between variables with a significant influence on hospitalization expenses.

The results showed that there was no significant difference in hospitalization expenses in terms of sex (*P* = 0.064), medical payment method (*P* = 0.223) and discharge method (*P* = 0.156). However, the hospitalization expenses of CD patients significantly influenced by marital status, age, admission route, length of stay, comorbidites, main treatment methods and readmission plan within 31 days of discharge (*P* < 0.05). The hospitalization expenses of patients were positively correlated with age and length of stay. The expenses of patients over 60 years old were the highest (median: 9,460 *Yuan*, IQR: 5,630–34,720 *Yuan*). The expenses of those who stayed for more than 20 days (median: 53,483 *Yuan*, IQR: 43,649–64,181 *Yuan*) were twice as high as those of patients who stayed <20 days. The hospitalization expenses of patients who were admitted to the emergency department (median: 8,970 *Yuan*, IQR: 5,848–24,930 *Yuan*), with comorbidities (median: 7,750 *Yuan*, IQR: 4,735–14,788 *Yuan*), and without a 31-day readmission plan (median: 7,655 *Yuan*, IQR: 4,452–16,786 *Yuan*) were higher than those of other conditions. The hospitalization expenses of surgical treatment was the highest (median: 40,924 *Yuan*, IQR: 16,510–51,627 *Yuan*), followed by procedural treatment (median: 7,493 *Yuan*, IQR: 5,245–11,348 *Yuan*). Medical treatment had the lowest expenses (median: 3,419 *Yuan*, IQR: 1,921–5,676 *Yuan*).

### Influencing factors of hospitalization expenses based on LASSO-LMM

**Figure 2:**
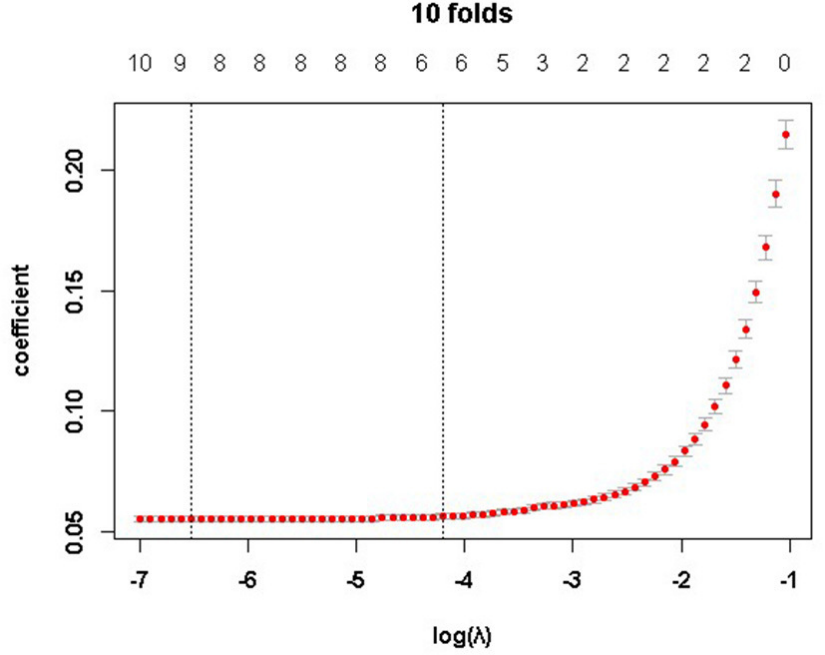
Penalty parameter (λ) selection and model error.

**Figure 3:**
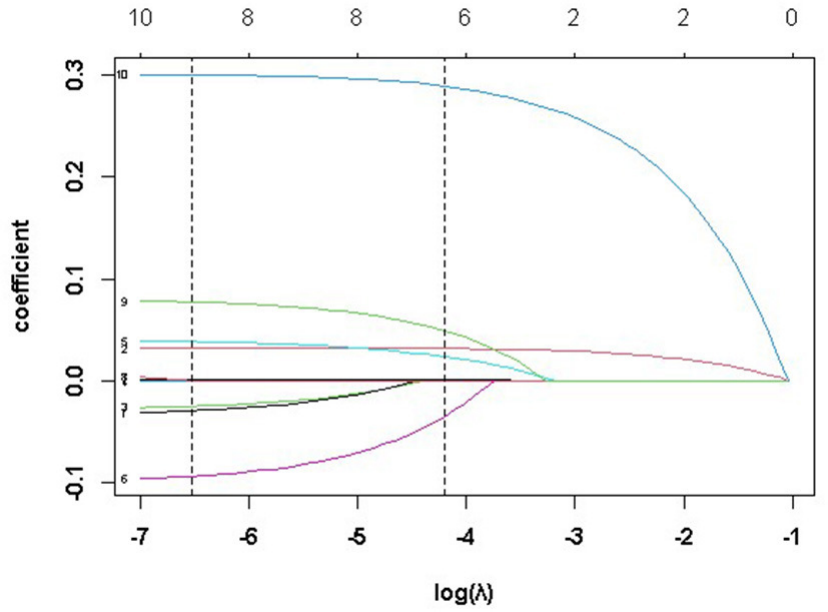
Penalty parameter (λ) and coefficient selection.

The optimal penalty parameter λ of the LASSO-LMM was determined by the 10-fold cross-validation method, and the values of lambda.min and lambda.1se were 0.001467 and 0.015013, respectively. We chose the lammda.1se as the optimal parameter of the model, and the six variables including age, length of hospital stay, marital status, admission route, comorbidities, and treatment methods were used as the influencing factors of CD hospitalization costs. The regression coefficients of all the other factors were 0. The changes in λ and the coefficients are presented in Figures 2, 3.

We fitted the LASSO-LMM model with marital status and year as random effects and the remaining five variables as fixed effects. The AIC and BIC of LASSO-LMM model were 319.033 and 306.241, respectively. As shown in Table 3, mode of treatment was the most important factor affecting hospitalization expenses. Compared with medical treatment, the coefficients of surgical treatment and procedural treatment on hospitalization expenses were 0.602 and 0.279, respectively. Patients who were older, had longer hospitalization days, and had comorbidities had higher hospitalization expenses, with coefficients of 0.002, 0.033, and 0.058, respectively. The hospitalization expenses of outpatients were lower than those of emergency patients.

**Table 3:** Summary of the multivariable regression analyses for hospital expenses in CD patients.

**Independent variables**	**Coefficient**	***SE***	***df***	***t*-value**	***P*-value**	**95%CI**	
						**Lower**	**Upper**
Intercept	3.366	0.040	710	83.413	<0.001	3.287	3.445
Age	0.002	0.0003	3,995	4.860	<0.001	0.001	0.002
Length of hospital stay	0.033	0.001	4,428	47.632	<0.001	0.031	0.034
Admission route (reference = emergency)	−0.087	0.016	4,424	−5.394	<0.001	−0.118	−0.055
Comorbidities (reference = no)	0.058	0.009	4,430	6.504	<0.001	0.041	0.076
**Treatment methods (reference** **=** **medical treatment)**
Surgical treatment	0.602	0.012	4,425	49.181	<0.001	0.578	0.626
Procedural treatment	0.279	0.009	4,426	31.633	<0.001	0.262	0.296

## Discussion

The prevalence of CD has been increasing worldwide. CD is a chronic and potentially disabling disease, commonly results in hospitalization, decreased quality of life and inability to work. It inevitably generates an increasing economic burden on health care systems, as CD is incurable and generally requires lifelong care and medication. Thus, it is imperative to explore the direct economic burden of CD to recognize its economic impact on health care systems and patients. Further, it is important to make decisions on health resource allocation to combat CD's economic impact. Our study provided a detailed description of the influencing factors of hospitalization expenses for CD patients in China. Linear mixed-effects models with a LASSO method were used to identify the key influencing factors associated with CD hospitalization expenses. We found that the hospitalization expenses of CD patients were related to the six factors, that are, age, length of hospital stay, marital status, admission route, comorbidities, and treatment methods.

Over the past 20 years due to the widespread availability of biological therapy, surgery rates for CD have declined. Nevertheless, approximately 30–40% of patients with CD still inevitably require surgery at some point in their lifetime (22). During patients' course of CD, their inflammatory activity periods alternate with remission periods. Whether biological treatment can modify the course of the disease and surgical rate of CD patients remains unproven. A registry study from Canada in 2019 showed that the introduction of biological therapy had not yielded the expected reductions in the rates of CD-associated hospitalizations and/or intestinal resections (23). Drug therapy and surgical procedures should be considered as equally viable treatment strategies (24).

CD leads to a considerable burden on health care systems due to its medical and surgical treatment. Our study showed that the hospitalization expenses of CD patients were closely related to the chosen treatment method, for which the expenses of surgical treatment was the highest, followed by procedural treatments. Based on the natural course of CD, the possibility of surgery increases over time. Hence, surgical or procedural therapy is still required for a proportion of patients with refractory or associated complications. As a result, the additional surgical and procedural treatments lead to increased hospitalization expenses. A prospective community-based study found that the total expenditure of surgery and procedures accounted for 31% of total expenditure (25). It showed that while the amount of surgery-related health care costs for the treatment of CD has declined, surgery costs remain an important factor in the cost of hospitalization for patients.

Our study shows that comorbidities were another important factor affecting the expenses of CD hospitalization. The presence of comorbidities in IBD patients had been proven to adversely affect their health-related quality of life, prolong their hospital stay and increase the risk of postoperative complications (26). Possibly due to common pathogenic pathways with other immune-mediated inflammatory diseases, CD has been associated with multiple chronic comorbidities such as bone diseases, cardiovascular diseases, iron-deficiency anemia, pain, psychological disorders and respiratory illness. It was found that persons with IBD have a more comorbidities burden than persons without IBD (27). A population-based registry study in Denmark also emphasized that some immune-mediated diseases (IMDs) and CD may have overlapping pathogenic pathways and that IMDs are usually more common in CD patients (28). The impact of CD comorbidities on medical practice and medical costs clearly cannot be ignored. Related diseases can alter or confuse the clinical manifestations and activity of CD, possibly affecting the disease's prognosis. Moreover, drug therapy, which has lower expenses than surgical procedural treatment, may be limited in CD patients with comorbidities. Building multidisciplinary teams to empower patient care is especially important for these patients (29). This means that they have more complex medical activities and increased care costs compared to other patients without comorbidities. A cross-sectional study conducted in Switzerland showed that compared with the non-IBD population, common comorbidities for IBD patients can more than double total costs (30). Our findings also suggest that the high medical costs caused by the comorbidities of CD patients deserve further investigation.

Admission through the emergency department and prolongation of hospital stay were independent predictors of higher hospitalization expenses for CD patients. Despite the current therapeutic arsenal for the treatment of CD, some patients with CD complications (6–16%) show acute complications and require emergency treatment (31). Acute complications that may occur in patients with CD include acute severe colitis, toxic megacolon, uncontrolled bleeding, perforation, abscess, intestinal obstruction and others. When patients are admitted through the emergency department, more urgent laboratory tests, imaging tests, and colonoscopies are required to assess their condition. In life-threatening situations, abscess drainage, colectomy, or total parenteral nutrition may be considered, which would result in additional resource consumption and increased healthcare costs.

The results of this study indicate that the hospitalization expenses of CD patients are positively correlated with age (*r*_*s*_= 0.127, *P* < 0.001) and length of hospital stay (*r*_*s*_= 0.804, *P* < 0.001). Patient age was weakly associated with hospitalization expenses. The pairwise comparison results of the age groups in Table 2 suggest that there was no significant difference in the hospitalization expenses of CD patients under 40 years old, while there existed differences in age groups above this age. The epidemiology, clinical characteristics, phenotype, disease course, cancer risks, therapeutic and monitoring strategy of CD patients are vary significantly by age of onset. This may lead to heterogeneity among CD hospitalization costs, highlighting the need for further research on hospitalization costs in CD patients of different ages. Length of hospital stay was strongly associated with hospitalization expenses. For CD patients, longer hospital stays often reflect disease severity or a lack of response to medication. The presence of IBD-specific complications involving malnutrition, the need for total parenteral nutrition, non-elective operation, clostridium difficile infection, venous thromboembolism, and hospital-acquired infections have all been shown to result in prolonged hospital stays (32). All of these situations require more nursing and medical support, which results in greater medical costs. Consequently shortening the length of hospital stay was considered to be one of the key strategies to mitigate healthcare financial stress and improve patient outcomes (33).

In our study, sex, discharge method, and medical payment method had no significant effect on the hospitalization expenses of CD patients. The Chinese government has achieved full coverage on the basic medical insurance for citizens, including the rural new cooperative medical scheme, urban resident-based basic medical insurance scheme and urban employee-based basic medical insurance scheme (34). Some biological agents used in the treatment of CD, such as infliximab, have been admitted into the reimbursement list of basic medical insurance in China. Therefore, medical payment method was not a factor affecting CD hospitalization expenses. While the results of the univariate analysis in Table 1 show that hospitalization expenses without readmission plans within 31 days after discharge are higher than those with planned readmissions, the multivariate analysis of the LASSO-LMM model in Table 3 indicates that the 31-day readmission plan factor has no significant effect on the hospitalization expenses of CD patients after controlling for other factors. In general, CD flare-ups, infections, or complications from unplanned operations during hospitalization are the most common reasons for readmissions (35). Hospital readmissions are costly to the national healthcare system. Especially, in the context of the implementation of DRG payment in China, reducing the unplanned readmission rate has become an important aspect of increasing the quality of healthcare services and decreasing the cost of medical services for patients with chronic diseases like CD.

This study has several limitations. First, we only described the medical expenses of two hospitals, and we were unable to assess health care received outside of our centers, so medical expense may be potentially underestimated. Second, our study only included data from the homepage of the medical records. The data have limited independent variables. Future research needs to incorporate more variables to establish better predictive models and to elucidate the impact of hospitalization expenses in CD patients. Lastly, our sample data were obtained from only two tertiary first-class hospitals. A larger sample size including patients from multiple different hospitals would further enhance the precision of estimates generated.

## Conclusion

Our study identified multiple factors that influence hospitalization expenses for CD patients. The degree of each influencing factor was different, with treatment mode being the most important impact on CD hospitalization expenses. Identifying and examining such factors can help to control healthcare expenditures. In the context of the current trial of DRG payment in China, our model can propose effective strategies for medical security departments to consider implementing personalized and precise hospitalization expense compensation scheme in the future. With all the above noted, reducing and/or controlling the hospitalization expenses of CD patients is bound to be a complex process that requires collaborative efforts. These urgently need to be addressed by medical security departments, hospital and patients themselves. It will be beneficial to reduce the economic burden of patients and the pressure of medical insurance fund through the medical security department formulating reasonable compensation programs, medical institutions providing optimal therapeutic regimens, and patients accepting treatment and nursing.

## Data availability statement

The raw data supporting the conclusions of this article will be made available by the authors, without undue reservation.

## Ethics statement

The studies involving human participants were reviewed and approved by the Ethics Committee of Sir Run Run Shaw Hospital, Zhejiang University school of Medicine and the Ethics Committee of the First Affiliated Hospital of Zhejiang Chinese Medical University. The Ethics Committee waived the requirement of written informed consent for participation.

## Author contributions

Conceptualization and funding acquisition: LW and ZL. Data curation: PZ. Methodology: LW and LL. Project administration and writing—review and editing: ZL. Software: LL. Writing—original draft: LW. All authors have read and approved the final manuscript.

## Funding

This research was funded by Zhejiang Provincial Natural Science Foundation of China (No. LQ20G030015), Health Commission of Zhejiang Province (No. 2020KY201), and Zhejiang Pharmaceutical Association (No. 2022ZYJ14).

## Conflict of interest

The authors declare that the research was conducted in the absence of any commercial or financial relationships that could be construed as a potential conflict of interest.

## Publisher's note

All claims expressed in this article are solely those of the authors and do not necessarily represent those of their affiliated organizations, or those of the publisher, the editors and the reviewers. Any product that may be evaluated in this article, or claim that may be made by its manufacturer, is not guaranteed or endorsed by the publisher.
